# The Effects of Geometry Size and Initial Microstructure on Deformation Behavior of Electrically-Assisted Micro-Compression in Ti-6Al-4V Alloy

**DOI:** 10.3390/ma15051656

**Published:** 2022-02-23

**Authors:** Jianxing Bao, Shoudan Lv, Bo Wang, Debin Shan, Bin Guo, Jie Xu

**Affiliations:** 1 Key Laboratory of Micro-Systems and Micro-Structures Manufacturing of Ministry of Education, Harbin Institute of Technology, Harbin 150080, China; jxbbao@126.com (J.B.); lvshd@hit.edu.cn (S.L.); shandb@hit.edu.cn (D.S.); bguo@hit.edu.cn (B.G.); 2 School of Materials Science and Engineering, Harbin Institute of Technology, Harbin 150001, China; 3 Department of Technology Development, Beijing Spacecrafts, Beijing 100094, China; wangb529@126.com

**Keywords:** size effect, initial microstructure, electrically-assisted forming, titanium alloy

## Abstract

In this study, electrically-assisted micro-compression (EAMC) tests were conducted for cylindrical specimens of Ti-6Al-4V alloy with four geometric sizes and three initial microstructures. The result showed that the specimen temperature nonlinearly increased with the square of current density. The quasi-static heat equilibrium equation was established to quantify the effects of the scale factor on the Joule heat temperature. Moreover, it was demonstrated that the Joule temperature scale effect had a greater effect on the flow stress than the sample size effect for specimens of different dimensions. It was noted that the 0.5 mm diameter sample displayed abnormal deformation behavior, which was related to surface oxidation leading a brittle surface layer. By comparison of the microstructures, it was found that the α→β phase transformation occured below the β transus temperature, which was attributed to the local Joule heat effect and the scattering of drift electrons during EAMC. Furthermore, the flow curves showed a strong dependence of the strength and ductility on the initial microstructure. The widmannstatten microstructure exhibited higher strength, smaller hardening rate and more easy flow localization compared with basket-weave microstructures, which was attributed to the low β phase content and narrow interlamellar spacing of α lamellae grains in the widmannstatten microstructure.

## 1. Introduction

In recent years, people are paying more attention to means of energy conservation and environmental protection due to the pressures of promoting growth while saving resources and protecting the environment. Electrically-assisted forming (EAF) has shown great potential in heating efficiency, environmental friendliness, increasing formability and optimizing microstructures. EAF, as a novel forming technology, applies a continuous or impulse current to workpieces during the forming process. When the electric current passes through materials, the plasticity of materials is improved and the forming force is significantly reduced, which is called the electroplastic effect (EPE) [1,2,3]. According to the research, the existence of the EPE has the ability to eliminate springback, promote recrystallization and weld microcracks [4,5,6]. So far, EAF technology has been widely used in processes of drawing, bending, blanking, rolling, stamping, incremental forming, friction stir welding, etc [7,8,9,10,11,12].

Titanium alloys are widely used in the fields of aerospace, weaponry and biomedical engineering due to their high strength, low density and anti-corrosion safety. However, the micro-forming of titanium alloys is a significant challenge in the micro-manufacturing field due to the low controllability of the shape and properties of materials under traditional hot working techniques. Furthermore, when the specimen size is scaled down to the mesoscale, the flow stress and friction behavior of materials become different from those of the macroscale one due to the so-called size effects [13,14], and the experience of the traditional forming process is no longer applicable in the field of micro-forming. The electrically-assisted micro-forming (EAMF) technology offers a promising prospect to solve these problems. The electric current passing through the titanium alloys will produce a strong thermal effect owing to the high resistivity, which makes it possible to quickly and efficiently to reduce the forming force of mesoscale forming [15,16,17,18]. It also offers also a way to weaken the size effect in plastic micro-forming by applying EPE. Previously, researchers focused on the effects of grain size on the deformation behavior during EAMF, and numerous composite material models were established [19,20,21,22]. However, the effects of the geometric dimensions on EAMF behavior of titanium alloys have been little investigated.

Typical microstructures of Ti-6Al-4V alloy can be equiaxial, bimodal, basket-weave, widmannstatten and martensitic. It is worth noting that the initial microstructures have an important effect on the hot working behavior of titanium alloys [23,24,25,26]. Zhang et al. [23] investigated the hot deformation behavior of Ti-6Al-4V alloy with transformed β microstructure, bimodal microstructure and martensitic microstructure through compression deformation, which revealed that the martensitic microstructure was more beneficial for achieving grain refinement. Lin et al. [27] discovered that the fracture characteristics of Ti-6Al-4V alloy were sensitive to the initial microstructures. The equiaxed α phases can prevent the formation and coalescence of microvoids, which was beneficial to improve the ductility. Our previous studies [28,29] have also shown that the shear banding evolution and microcracks nucleation were directly influenced by the initial microstructures of Ti-6Al-4V alloy, so it is important to distinguish the effect of initial microstructures on EAMF of Ti-6Al-4V alloy.

Electrically-assisted micro-forming has shown great potential in increasing formability and optimizing microstructures. However, the effects of initial microstructure and geometric dimension on the micro-deformation behavior in Ti-6Al-4V alloys have been little investigated. For this purpose, the EAMC tests were conducted for cylindrical specimens of Ti-6Al-4V alloy with four geometric dimensions and three initial microstructures in this study. The true stress-strain curves and strain hardening rate were recorded and compared. Furthermore, for better understand the deformation behaviors, the microstructure evolution and surface morphology were studied by optical microscopy (OM), field emission scanning microscopy (FE-SEM) and transmission electron microscopy (TEM).

## 2. Materials and Methods

### 2.1. Specimen Preparation

**Figure 1:**
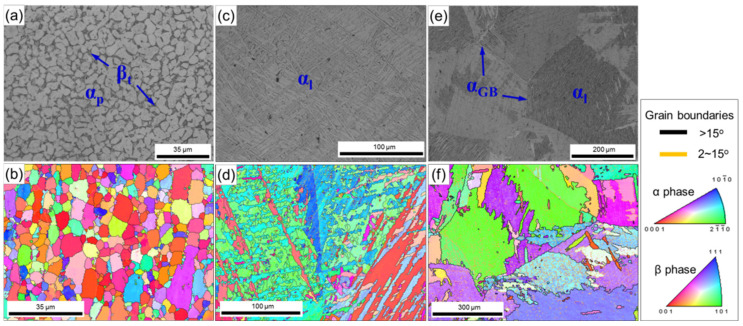
Initial microstructures of Ti-6Al-4V alloy: (**a**,**b**) equiaxed microstructure, (**c**,**d**) basket-weave microstructure and (**e**,**f**) widmannstatten microstructure.

Commercial drawn Ti-6Al-4V alloy rods having a diameter of 10 mm and length of 70 mm were selected as the as-received materials in this study. The composition of the raw alloy is 5.9 aluminum, 3.9 vanadium, 0.01 iron, 0.13 oxygen, 0.02 carbon, 0.03 nitrogen, and 0.015 hydrogen (all in wt%), the balance being titanium. The raw material was annealed at temperatures of 900 °C, 980 °C, 1050 °C for 1 h and cooled in air to obtain the equiaxed, basket-weave and widmannstatten microstructures, respectively. As shown in Figure 1, the average size of equiaxed α is ~8 μm and approximately 92% primary equiaxed α are distributed in transformed β matrix for the equiaxed microstructure. The basket-weave microstructure comprises α lamellae of about 0.5~8 μm and β phases spread over the boundaries of α phases. The widmannstatten microstructure consists of the coarse prior β grains, in which the α lamellae is distributed among the transformed β matrix. The cylindrical specimens with diameter of 0.5, 1, 1.5, and 2 mm and height-diameter ratio of 1.5 were prepared via precision machining process. In order to ensure that the electric current can pass through the specimen, all specimens were cleaned with acetone to remove any dirt and oil on the surface.

### 2.2. EAMC Tests and Microstructure Characterization

**Figure 2:**
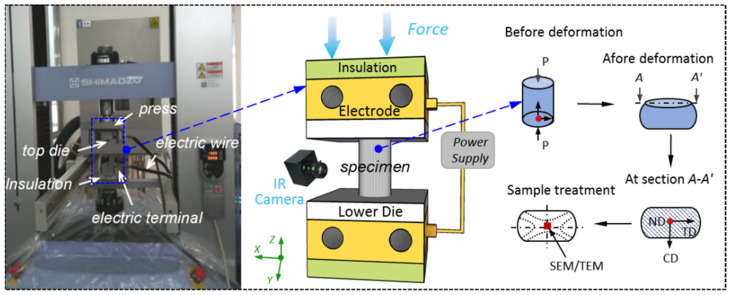
Schematic diagram of the electrically-assisted forming platform and the micro-compression samples at different stages in the experimental process.

The EAMC experiments were carried out on an electrically-assisted forming platform. As shown in Figure 2, it comprises a universal materials tester (AG-X 50kN, Shimadzu Corp., Kyoto, Japan), power supply (MicroStar CRS-LFP20-500, Dynatronix Inc., Wisconsin, WI, USA), hard alloy dies and mica insulation plates. The power supply has a maximum output current of 500 A and a maximum voltage of 20 V. A infrared thermal imager (T660, FLIR Systems, Inc., Wilsonville, OR, USA) was used to monitor the temperature of specimen during the EAMC tests. The thermal resolution and temperature range are 1 °C and −40~2000 °C respectively. The temperature measurement accuracy is greatly affected by the surface emission rate of materials when infrared thermal imager is used. In order to keep the specimen surface with high and consistent emissivity, the black heat-resistant lacquer was sprayed on the surface of specimens. The emissivity of the painted surface is 0.85 after calibration by thermoelectric couple.

In EAMC tests, different current densities (30, 40, 50 and 70 A/mm^2^) were first applied to the specimens. The temperature of specimen will rise rapidly owing to the Joule heat effect, and the compression tests will begin after the temperature remains unchanged. The specimens were compressed with a fixed height reduction of 60% or until fracture. Also, it should be mentioned that the current passing through the specimen was kept constant during EAMC tests. After the compression, the power supply was turned off and the dies were recovered to room temperature before proceeding to the next experiment. Each experiment was repeated three times to ensure reproducibility and the strain rate used in this study was 0.01 s^−1^.

The microstructure evolution and surface topography of specimens were observed by OM (GX71, OLYMPUS, Kyoto, Japan), FE-SEM (Quanta 200F FEI, Hillsboro, OR, USA), electron back scatter diffraction (EBSD, FEI Quanta 200F) with TSL OIM analysis software, and TEM (FEI Talos F200x). The specimens for OM were etched in a solution that contained 2 mL hydrofluoric acid, 1 mL nitric acid and 17 mL water after mechanical polishing. The specimens for EBSD observations were electropolished in a solution of 60% carbinol, 34% *n*-butanol and 6% perchloric acid with a current of 70 mA at no greater than −25 °C. The TEM foils were prepared by the ion milling method after mechanical grinding. X-ray diffraction (XRD) was performed using an X’Pert Pro MPD system (PANalytical B.V., Almelo, Netherlands) with Cu-Kα radiation having a wavelength of 1.5405 Å.

## 3. Results and Discussion

### 3.1. Geometry Size Effects of Joule Heat Temperature

**Figure 3:**
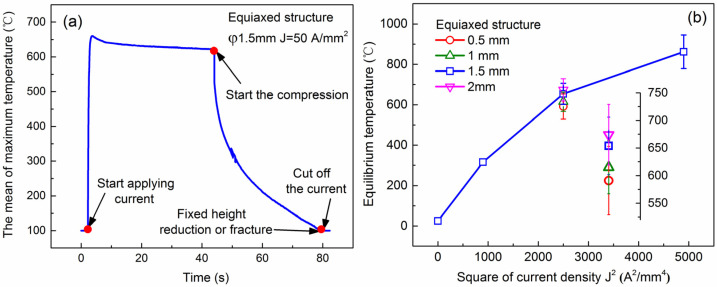
(**a**) The mean of maximum temperature history of equiaxed microstructure specimen with 1.5 mm in diameter under 50 A/mm^2^ and (**b**) the relationship between the equilibrium temperature and square of current density of equiaxed microstructure specimen with different specimen sizes.

In the process of EAMC, the current through the specimen remains constant, but the cross-sectional area will increase with the decrease of the specimen height. And these will lead to continuous decrease of the actual current density during EAMC. Thus, the nominal current density, that is initial current intensity/initial cross-section area, was used in the study. The variation of maximum temperature of equiaxed microstructure specimen under 50 A/mm^2^ with time is shown in Figure 3a. It can be seen that the temperature of specimen increases rapidly from room temperature to an equilibrium temperature. Then it decreases significantly with time during micro-compression, which is attributed to the decrease of current density caused by the increase of the cross-sectional area of the sample. Figure 3b displays the relationship between the equilibrium temperature and square of current density for the specimen with diameter of 1.5 mm. The equilibrium temperature is 316 ± 16 °C, 654 ± 52 °C and 862 ± 8 °C corresponding to the current densities of 30, 50 and 70 A/mm^2^, respectively.

**Figure 4:**
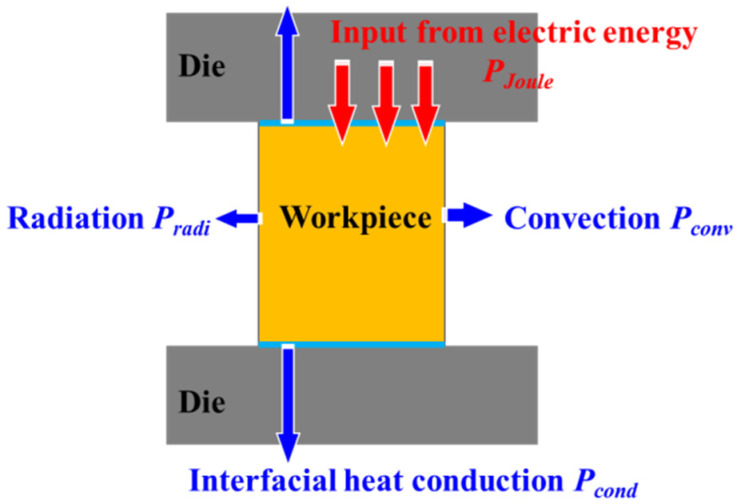
Schematic diagram of Joule heat model of EAMC specimen.

Moreover, it is observed that the equilibrium temperature nonlinearly increases with the square of current density. This may seem inconsistent with adiabatic Joule heating law. Therefore, the thermal conduction (*P_cond_*) between specimen and die, thermal convection (*P_conv_*) and thermal radiation (*P_radi_*) cannot be ignored during EAMC, as shown in Figure 4. In general, this state of thermal equilibrium is also known as quasi-static equilibrium. The quasi-static heat equilibrium equation of the specimen is obtained by Equation (1):

(1) $$P_{J}=P_{cond}+P_{conv}+P_{radi}$$

where:

(2) $$P_{J}=A_{s}\rho_{e}HJ^{2}$$

(3) $$P_{cond}=2A_{s}k(T-T_{f})$$

(4) $$P_{conv}=2\pi rHh(T-T_{R})$$

(5) $$P_{radi}=2rH\varepsilon \sigma \left(T^{4}-T_{f}^{4}\right)$$

*J*, *ρ_e_*, *T*, *T_R_*, *T_f_*, *H*, *r*, *A_s_* are the current density, electrical resistance, specimen temperature, room temperature, dies temperature, sample height, radius and sectional area of cylindrical sample, respectively. *k* is the thermal conductivity coefficient, *h* is the convection heat transfer coefficient, *σ* is the Boltzmann constant (*σ* = 1.38 × 10^−23^ J/K) and *ε* is the emissivity (*ε* = 0.83). Substituting Equations (2)–(5) into Equation (1), yields:

(6) $$A_{s}\rho_{e}HJ^{2}=2A_{s}k(T-T_{f})+2\pi rHh(T-T_{R})+2rH\varepsilon \sigma (T^{4}-T_{f}^{4})$$

It can be seen from Equation (6) that the temperature of the specimen will increase non-linearly with the increase of current density. The electrical resistivity, the thermal conductivity and the convection coefficient are obtained in our previous study [30]. After calculation, it is found that among the three heat losses, interface thermal conduction accounts for the largest proportion (about 82%), radiation thermal transfer accounts for the middle proportion (about 14%), and air convection thermal transfer accounts for the smallest proportion (about 4%).

Additionally, it can be found the maximum temperature increases with the increase of specimen size under 50 A/mm^2^ (Figure 3b insets), giving values such as 591 ± 62 °C for 0.5 mm diameter, 615 ± 48 °C for 1 mm diameter and 674 ± 55 °C for 2 mm diameter samples. This result demonstrates that the specimen scale has a remarkable influence on the current-induced Joule temperature, that is the scale effect of Joule heat temperature. By contrast, the heat loss of interface conduction term has a larger proportion during EAMC. Previous studies have found that the conduction term has the most significant impact in those three kinds of heat loss [21,30]. It indicates that the loss of interface conduction can be approximately as the total heat loss by an appropriate proportionality factor. Here *M* is defined as the proportionality factor. Thus Equation (6) is simplified to:

(7) $$\rho_{e}HJ^{2}=2Mk(T-T_{f})$$

If $\alpha =A_{s}^{2}/H$ is defined as the size factor:

(8) $$\rho_{e}I^{2}=2M\alpha k\left(T-T_{f}\right)$$

**Figure 5:**
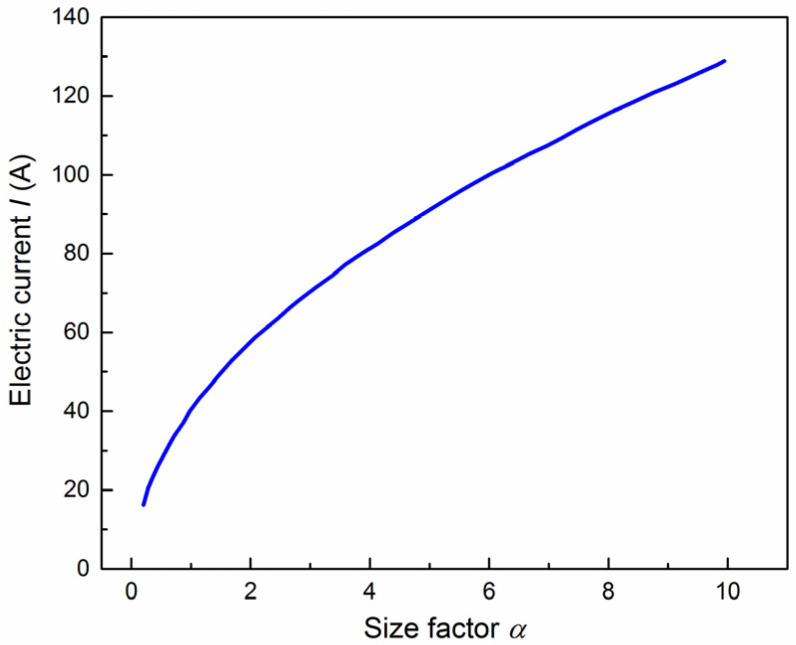
The relationship between electric current and size factor at the specimen temperature of 500 °C.

Figure 5 displays the relationship between electric current and size factor at the specimen temperature of 500 °C obtained by Equation (8). It can be seen that the electric current required to reach the target temperature will increase with the increase of the scale factor. The results are in good agreement with the experimental temperature values.

### 3.2. The Effects of Geometry Size on Deformation Behavior

**Figure 6:**
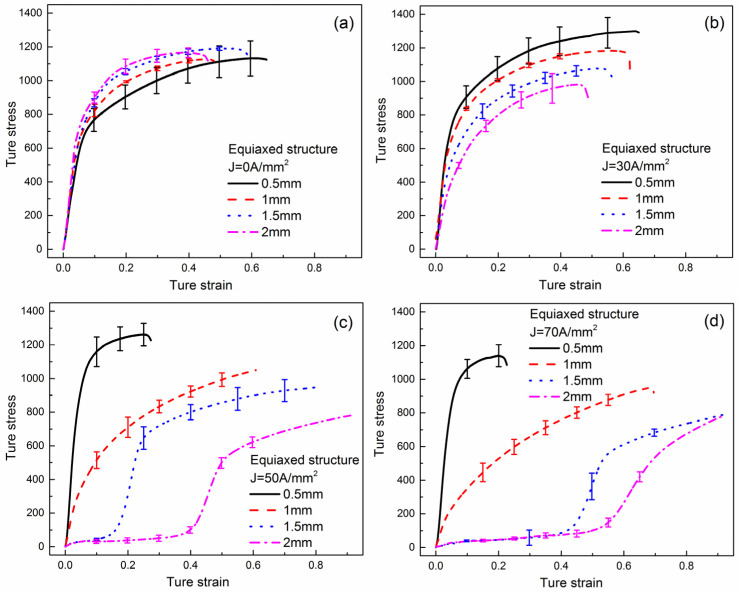
True stress-true strain curves of equiaxed microstructure specimen with different specimen sizes: (**a**) 0.5 mm, (**b**) 1 mm, (**c**) 1.5 mm, (**d**) 2 mm.

The true stress-true strain curves with varying geometric sizes of equiaxed microstructure specimen under different current density are shown in Figure 6. The results show that the true stress slightly decreases with the decrease of the specimen size under no-load current conditions. By contrast, the true stress decreases with the increase of the specimen size under load current, and the decreasing trend is more obvious that the increase of current density. This is attributed to Joule heat temperature increasing with the increase of specimen size. Therefore, the softening effect of Joule heating is more obvious in a larger sample size for the same current density. It is readily apparent that the sample size effect and the Joule temperature scale effect have the opposite effects on flow stress, and that the Joule temperature is a dominant factor. Furthermore, the specimen with diameter of 0.5 mm exhibits a significantly greater fracture strain than other specimens under no-load current conditions, while the fracture stain decreases gradually with increasing current density under load current conditions. This could be because of the overall deformation of small size specimen is more uniform at room temperature, but the high temperature causes severe surface oxidation and arc ablation in the samples with smaller size, leading to the hard and brittle stress-strain curves.

**Figure 7:**
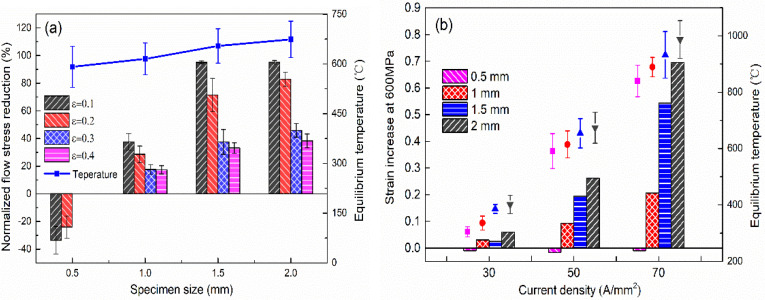
(**a**) Normalized flow stress reduction with different specimen sizes under 50 A/mm^2^ and (**b**) the strain increment with different specimen sizes under different current densities.

For further analysis of the effect of specimen size on the flow stress, Figure 7a shows the normalized flow stress reduction of different specimen sizes under 50 A/mm^2^ corresponding to the compression strain of *ε*_0.1_, *ε*_0.2_, *ε*_0.3_, and *ε*_0.4_. It can be clearly observed that the softening ability increases with the increase of specimen size, which reveals that the smaller size specimen needs larger current density to achieve the same softening degree. As an important indicator, the compressible deformation amount is used to evaluate the deformation of titanium alloy during EAMC. Figure 7b shows the relationship between strain increment and current density at *σ*_600_ with various specimen sizes. It can be seen that the compressible deformation amount of the specimen increases with the increase of current density. For example, the strain increment of the specimen with a diameter of 2 mm under 70 A/mm^2^ is 0.695 relative to room temperature. The strain increment increases with the increase of specimen size. When the current density is 70 A/mm^2^, the strain increment of the specimen with diameter of 2 mm is 1.28 times that of the specimen with a diameter of 1.5 mm and 3.37 times that of the specimen with a diameter of 1 mm. In addition, when the current density is 50 A/mm^2^, the compression increment of the specimen with a diameter of 0.5 mm dose not conform to the above deformation behavior. This is because the specimen will not only soften due to the Joule heat effect, but also harden due to the enhancement of hydrogen and oxygen absorption, and the softening effect is smaller than the hardening effect for the specimen with a diameter of 0.5 mm. This illustrates that the stress drop induced by Joule heat temperature has an obvious correlation with specimen geometric dimensions.

**Figure 8:**
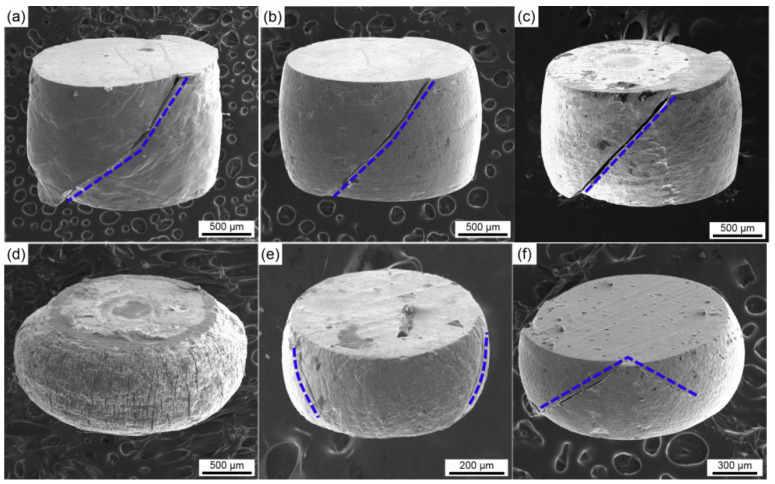
The surface morphology of the compressed equiaxed microstructure specimens under different specimen sizes and current densities: (**a**) 1.5 mm–0 A/mm^2^, (**b**) 1.5 mm–30 A/mm^2^, (**c**) 1.5 mm–50 A/mm^2^, (**d**) 1.5 mm–70 A/mm^2^, (**e**) 0.5 mm–50 A/mm^2^, (**f**) 1 mm–50 A/mm^2^.

The SEM images of surface topographies of the compressed specimens with equiaxed microstructure under different specimen sizes and current densities are shown in Figure 8. It can be seen that the compressed specimens show shear fracture when the applied current density is 0 and 30 A/mm^2^. This is a combined effect of friction and pressure, which leads to shear failure in the compression direction of 45°. When the current density is up to 70 A/mm^2^, the specimen did not fracture. The surface oxidation and arc ablation become more and more serious with the increase of current density. It is found that there are many small cracks on the side surface and serious arc ablation on the end surface of the specimen under 70 A/mm^2^ conditions. Moreover, the fracture direction of the sample appears random when the sample size is less than 1 mm at same current density. Since the specimen is very small, the charge can easily accumulate on the surface, and the titanium alloys tend to absorb hydrogen and oxygen, which leads to the brittleness of the surface layer. This result can be used to further explain the abnormal stress-strain curves of 0.5 mm specimen.

**Figure 9:**
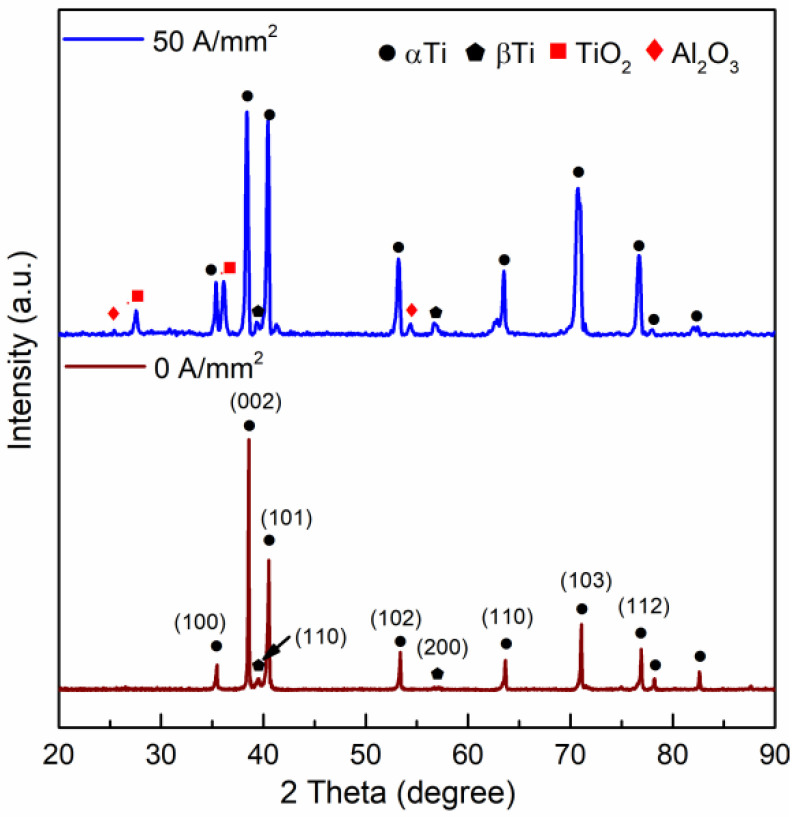
X-ray diffraction profiles of the compressed equiaxed microstructure specimens at different current densities.

To analyze the crystal structure and surface composition of compressed specimens, Figure 9 shows the XRD profiles of the compressed specimens with diameter of 1.5 mm under 50 A/mm^2^. In the 0 A/mm^2^ sample, it is readily apparent that only the α-phase Ti and β-phase Ti are visible. This is in good agreement with the standard diffraction peaks of the two-phase (α + β) TC4 titanium alloy. After EAMC, the diffraction peaks intensity of (200) for β-phase Ti becomes stronger. By the semi-qualitative analysis of XRD, it shows that the content of β-phase is much higher in the EAMC specimens. In addition, the diffraction peaks of TiO_2_ and Al_2_O_3_ are found on the surface of the EAMC specimens. This indicates the surface oxidation occurred during EAMC, which are consistent with the SEM analysis results.

**Figure 10:**
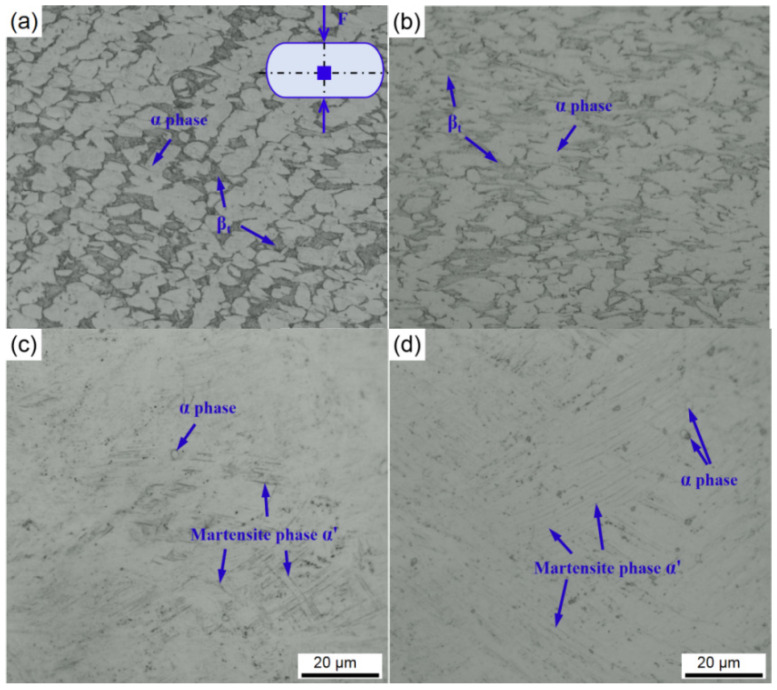
The microstructure of cross section for the compressed equiaxed microstructure specimens with a diameter of 1.5 mm at different current densities: (**a**) 0 A/mm^2^, (**b**) 30 A/mm^2^, (**c**) 50 A/mm^2^, (**d**) 70 A/mm^2^.

Figure 10 compares the microstructures of the specimens with a diameter of 1.5 mm after compression under different current densities. When the current density increases to 30 A/mm^2^, the equiaxed α phase is elongated, and β phase ratio is increased. For the specimen under 50 A/mm^2^, acicular martensite phase α′ is observed. It is well known the martensite phase occurs α′ in the β transus temperature above as well as rapid cooling rate [31]. Nevertheless, the maximum temperature of the specimen is only 654 ± 52 °C at 50 A/mm^2^, which is far lower than the β transus temperature over 900 °C for Ti-6Al-4V alloy in the conventional hot working process [32,33,34]. This can be caused by: (1) the micro sample has a large temperature gradient both the inside and outside; (2) local Joule heat effect can generate instantaneous local high temperature and thus accelerate the atom diffusion coefficient; (3) the scattering of drift electrons provides additional energy to lower the activation energy of atomic diffusion. Moreover, when the current density is increased to 70 A/mm^2^, the acicular martensite phase α′ has grown significantly due to the thermal effect of electric current. It is noteworthy that the microstructure distribution become complex under EAMC. Both martensite phase α′ and equiaxed α can exist simultaneously when the current density is greater than 50 A/mm^2^. This is attributing to the inhomogeneous internal temperature distribution the micro specimen during EAMC.

### 3.3. The Effects of Initial Microstructure on Deformation Behavior

**Figure 11:**
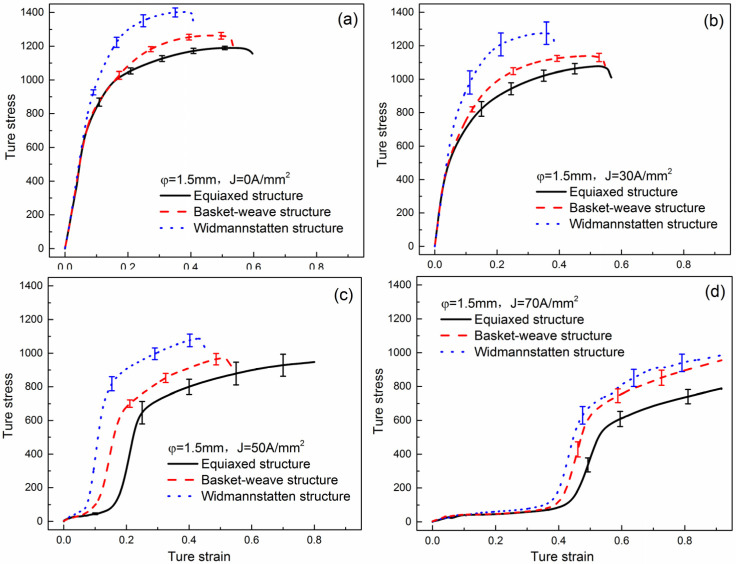
True stress-true strain curves of different initial microstructure specimens under varying current densities: (**a**) 0 A/mm^2^, (**b**) 30 A/mm^2^, (**c**) 50 A/mm^2^, (**d**) 70 A/mm^2^.

Figure 11 reveals the true stress-strain curves of compressed samples with equiaxed, basket-weave and widmannstatten microstructures under different current densities. It can be observed that the flow curves show strong dependence of initial microstructure on strength and ductility. The flow curves of the widmannstatten microstructure exhibit higher strength, while the curves show better ductility in equiaxed microstructure. This is because α phase with HCP crystal structure has a limited number of independent slip systems compared with β phase with BCC crystal structure. And the content of transformed β phase is the highest in equiaxed microstructure and the lowest in widmannstatten microstructure. On the other hand, the colonies in widmannstatten or basket-weave specimens have numerous α/β interfaces, and these α/β interfaces will act as a strong obstacle to the motion/slip transmission of dislocations [27,29,35]. Furthermore, the flow stress decreases with the increase of current density. The results show that Joule heat effect is a dominant factor to reduce the flow stress. Moreover, some stress-strain curves, especially at larger current density, exhibit a S-shaped flow, which may be related to local softening of samples during EAMC [30].

**Figure 12:**
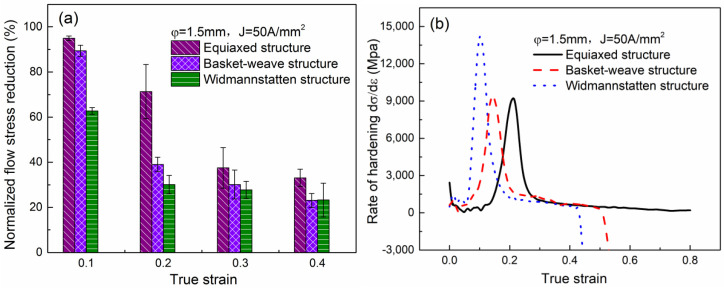
(**a**) Normalized flow stress reduction with different strain and (**b**) the relationship between strain hardening rate and strain under 50 A/mm^2^ for different initial microstructure specimens.

Figure 12a shows the flow stress drop corresponding to the compression strain of *ε*_0.1_, *ε*_0.2_, *ε*_0.3_ and *ε*_0.4_ for different initial microstructure specimens. The stress drop is the highest in the equiaxed microstructure and shows the minimum value in the widmannstatten microstructure. For example, when the strain is *ε*_0.1_, the average stress drop is about 94% and 63% for equiaxed and widmannstatten microstructures, respectively. This indicates that the electroplastic sensitivity of strength is prominent in the equiaxed specimen compared with other microstructures. The strain-hardening rates (dσ/dε) of different initial microstructure specimens is shown in Figure 12b. The peak strain hardening rate first appears at a low strain *ε*_0.02_ for the three samples. Whereafter the peak strain hardening rate decreased and shifted to the right accompanying with the microstructure change from widmannstatten through basket-weave to equiaxed. Such as the equiaxed microstructure specimen exhibits a peak hardening rate at relatively large strains (>0.2), whereas in widmannstatten microstructure specimen it is at relatively low strains (<0.1). It indicates that the softening effect of electric current is more obvious in equiaxed microstructure specimens. Finally the hardening rate values decrease gradually to 0 for basket-weave and widmannstatten microstructures, indicating that plastic instability occurs in the specimens.

**Figure 13:**
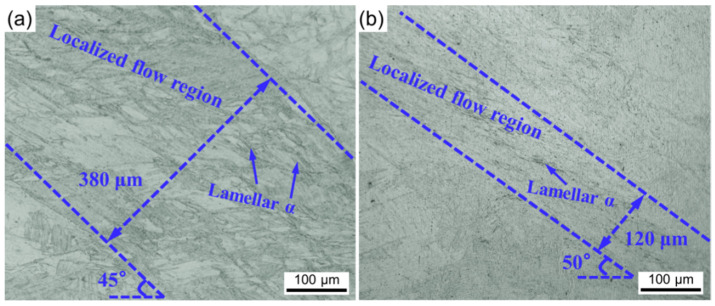
The microstructure of cross section of the compressed specimens for (**a**) basket-weave microstructure and (**b**) widmannstatten microstructure under 50 A/mm^2^.

The cross-sectional OM images of the basket-weave and widmannstatten microstructure compressed samples are shown in Figure 13. It can be seen the initial microstructures have a significant effect on the localized flow regions. The localized shear regions can be identified as the regions between two blue lines. The width is approximately 360 μm in the basket-weave microstructure and 120 μm in the widmannstatten microstructure, respectively. Furthermore, the localized flow regions tilt ~45° from CA in the basket-weave microstructure and ~50° in the widmannstatten microstructure. For the basket-weave microstructure, the localized flow regions contain many banded microstructures, in which α lamellae show a localized flow pathlines along the shear direction. In the widmannstatten microstructure, the bending α lamellae grains are very obvious. The bending of α lamellae is due to the intense shear in the narrow deformation zones. In fact, the difference of localized flow of the basket-weave and widmannstatten microstructures depend on the interlamellar spacing of the lamellae grains [29]. Thus, as shown by the above experimental results, the widmannstatten microstructure specimens exhibit higher strength, lower elongation, more easy flow localization and shear deformation due to the narrow interlamellar spacing of α lamellae grains.

**Figure 14:**
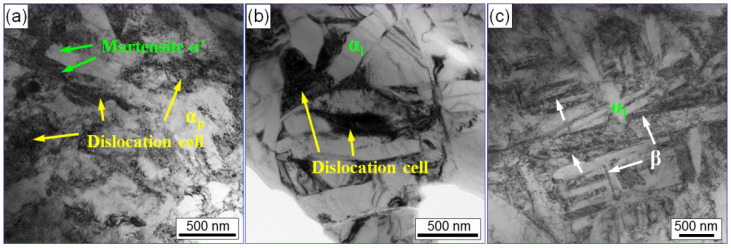
TEM images of the compressed specimens for (**a**) equiaxed microstructure, (**b**) basket-weave microstructure and (**c**) widmannstatten microstructure under 50 A/mm^2^.

The microstructures of the deformation zone are characterized further by TEM. Figure 14a shows the TEM images of the compressed specimens with equiaxed microstructure. It can be found the dislocation density is very high in equiaxed α_p_ and the microstructure is mainly composed of dislocation cells mainly. Moreover, there are some acicular martensite α′ grains in equiaxed α_p_ grains, which is a result of the local phase transformation. Figure 14b is the TEM image of the compressed specimens with basket-weave microstructure. It clearly exhibits that there are high-density dislocations in the β grains, while there are almost no dislocations in the lamellar α_l_ grains. Figure 14c is the TEM image of the compressed specimens with widmannstatten microstructure. Compared with the basket-weave microstructure, there is no significant difference in dislocation in β grains, while local dislocation pileup is found in α_l_ grains.

## 4. Conclusions

The effects of geometric size and initial microstructure on deformation behaviors during EAMC were studied using Ti-6Al-4V alloy cylindrical specimens. The main conclusions can summarized as follows:

(1) The quasi-static Joule heat temperature of specimens increases non-linearly with the square of current density during EAMC. The heat equilibrium equation was established to quantify the effect of specimen size on Joule heat temperature by introducing the scale factor.
(2) The Joule temperature scale effect has a greater effect on the flow stress than the sample size effect for specimens of different dimensions. The sample with a diameter of 0.5 mm displayed abnormal flow behavior, which is related to surface oxidation leading to formation of a brittle surface layer.
(3) Both the martensite phase α′ and equiaxed α can exist simultaneously in compressed equiaxed microstructure specimens. The α→β phase transformation occurs below the β transus temperature, which is attributed to the local Joule heat effect and the scattering of drift electrons during EAMC.
(4) Due to the low β phase content and narrow interlamellar spacing of α lamellae grains, specimens with widmannstatten microstructure exhibit higher strength, smaller hardening rate and more easy flow localization compared with basket-weave microstructures.

## Data Availability

The raw/processed data required to reproduce these findings cannot be shared at this time as the data also forms part of an ongoing study.
